# TRPV1 Is a Potential Tumor Suppressor for Its Negative Association with Tumor Proliferation and Positive Association with Antitumor Immune Responses in Pan-Cancer

**DOI:** 10.1155/2022/6964550

**Published:** 2022-10-18

**Authors:** Rongfang Nie, Qian Liu, Xiaosheng Wang

**Affiliations:** ^1^Biomedical Informatics Research Lab, School of Basic Medicine and Clinical Pharmacy, China Pharmaceutical University, Nanjing 211198, China; ^2^Cancer Genomics Research Center, School of Basic Medicine and Clinical Pharmacy, China Pharmaceutical University, Nanjing 211198, China; ^3^Big Data Research Institute, China Pharmaceutical University, Nanjing 211198, China

## Abstract

**Background:**

Although numerous studies have shown that the expression and activation of TRPV1 have an important role in cancer development, a comprehensive exploration of associations between *TRPV1* expression and tumor proliferation, microenvironment, and clinical outcomes in pan-cancer remains insufficient.

**Methods:**

From The Cancer Genome Atlas (TCGA) program, we downloaded multiomics data of ten cancer cohorts and investigated the correlations between *TRPV1* expression and immune signatures' enrichment, stromal content, genomic features, oncogenic signaling, and clinical features in these cancer cohorts and pan-cancer.

**Results:**

Elevated expression of *TRPV1* correlated with better clinical outcomes in pan-cancer and diverse cancer types. In multiple cancer types, *TRPV1* expression correlated negatively with the expression of tumor proliferation marker genes (*MKI67* and *RACGAP1*), proliferation scores, cell cycle scores, stemness scores, epithelial-mesenchymal transition scores, oncogenic pathways' enrichment, tumor immunosuppressive signals, intratumor heterogeneity, homologous recombination deficiency, tumor mutation burden, and stromal content. Moreover, *TRPV1* expression was downregulated in late-stage versus early-stage tumors. In breast cancer, bladder cancer, and low-grade glioma, *TRPV1* expression was more inferior in invasive than in noninvasive subtypes. Pathway analysis showed that the enrichment of cancer-associated pathways correlated inversely with *TRPV1* expression levels.

**Conclusion:**

*TRPV1* upregulation correlates with decreased tumor proliferation, tumor driver gene expression, genomic instability, and tumor immunosuppressive signals in various cancers. Our results provide new understanding of the role of TRPV1 in both cancer biology and clinical practice.

## 1. Introduction

Ion channels are important in modulating a variety of biological processes, such as intracellular calcium (Ca^2+^) functioning in regulating cell motility, cell cycle, and apoptosis [1] and potassium (K^+^) channels modifying cell proliferation, cell migration, invasion, and apoptosis [2]. Ion channels are potential pharmacological targets for cancer treatment for their essential roles in tumor development, proliferation, and invasion [3, 4]. The nonselective cation channel TRPV1 (transient receptor potential cation channel subfamily V member 1) plays significant roles in cancer onset and advancement [5, 6]. TRPV1 was first discovered in 1997 and was defined as a pain and heat receptor [7]. It can be activated by a variety of factors, including capsaicin [7], lipopolysaccharides [8], vanilloids [9], heat [9], protons [9], phosphoinositide 4,5-bisphosphate [10], vitamin D [11], and Toll-like receptor 4 [12]. TRPV1 is also activated in a variety of cancers, such as tongue squamous cell cancer [13], pancreatic cancer [14], breast cancer [15], and prostate cancer [16]. Nevertheless, some studies have revealed a tumor suppressor role of TRPV1 in various cancers. For example, TRPV1 activation can reduce glioma expansion and prolong survival of glioma patients [17]. TRPV1 can inhibit the development of gastric cancer, and its downregulation is associated with poor survival in gastric cancer [18]. TRPV1 can downregulate EGFR levels by inducing EGFR ubiquitination and degradation, thereby inhibiting the EGFR/MAPK signaling in pancreatic cancer cells [19]. In clear cell renal cell carcinoma (ccRCC), *TRPV1* expression is associated with immune infiltration and inhibits the progression of ccRCC [20]. The expression and activation of TRPV1 can activate protein tyrosine phosphatase 1B (PTP1B) to inhibit EGFR-associated intestinal tumorigenesis [21]. TRPV1 overexpression can activate p53 and induce apoptosis to inhibit tumor proliferation in melanoma [22].

Although these prior studies have revealed the diverse roles of TRPV1 in cancer, a systematic investigation of its associations with various clinical and molecular features in pan-cancer remains insufficient. To fill this research gap, we explored correlations of *TRPV1* expression with immune signatures' enrichment, progression phenotypes, and clinical outcomes in ten cancer types from The Cancer Genome Atlas (TCGA) program. This study is aimed at furnishing novel insights into the role of TRPV1 in both cancer biology and clinical practice.

## 2. Materials and Methods

### 2.1. Datasets

We downloaded the data of transcriptomes (RSEM normalized), somatic mutations, and clinical information for ten TCGA cancer cohorts from the GDC database (https://portal.gdc.cancer.gov/). All gene expression values were transformed by log_2_(*x* + 1) before subsequent analyses. The ten cancer types included brain lower grade glioma (LGG), head and neck squamous cell carcinoma (HNSC), lung adenocarcinoma (LUAD), breast invasive carcinoma (BRCA), stomach adenocarcinoma (STAD), pancreatic adenocarcinoma (PAAD), liver hepatocellular carcinoma (LIHC), bladder urothelial carcinoma (BLCA), cervical squamous cell carcinoma and endocervical adenocarcinoma (CESC), and skin cutaneous melanoma (SKCM). We summarized these datasets in Supplementary Table S1. We also downloaded subtype-related data from TCGA with the R function “TCGAquery_subtype” in the R package “TCGAbiolinks” [23].

### 2.2. Calculation of the Enrichment Scores of Immune-Related Signatures, Phenotypes, and Cancer-Related Pathways

We calculated the enrichment score of an immune-related signature, tumor phenotype, or cancer-related pathway in a tumor sample by the ssGSEA algorithm [24] based on the expression profiles of their marker or pathway gene sets. We presented these gene sets in Supplementary Table S2.

### 2.3. Quantification of Tumor Purity and Stromal Components

We quantified tumor purity and stromal components for each tumor with the ESTIMATE algorithm [25] with the input of gene expression profiles.

### 2.4. Quantification of Tumor Mutation Burden (TMB), Homologous Recombination Deficiency (HRD), and Intratumor Heterogeneity (ITH)

A tumor's TMB was defined as its total number of somatic mutations. The HRD scores of TCGA cancers were obtained from a previous publication [26]. We used the DEPTH algorithm [27] to evaluate ITH.

### 2.5. Survival Analysis

We compared the survival rates between different subgroups of cancer patients. A total of four endpoints were compared, including overall survival (OS), disease-free survival (DFS), disease-specific survival (DSS), and progression-free interval (PFI). We plotted the Kaplan−Meier survival curves to exhibit the differences in survival rates. The log-rank test was used to assess the significance of survival differences. We utilized the R package “survival” to perform survival analyses.

### 2.6. Identification of an Interaction Network of TRPV1

The interaction network of TRPV1 was identified by BioGRID [27] with the default parameters in the tool.

### 2.7. Pathway Analysis

Based on *TRPV1* expression levels, we defined the high-*TRPV1*-expression-level (upper third) and low-*TRPV1*-expression-level (bottom third) subgroups in pan-cancer. We first identified differentially expressed genes with a threshold of fold change (FC) > 1.5 and the false discovery rate (FDR) < 0.05. We then selected the 500 upregulated genes in high-*TRPV1*-expression-level tumors and the 500 upregulated genes in low-*TRPV1*-expression-level tumors with the smallest FDRs. We input both sets of genes into the GSEA web tool [26] to identify the pathways significantly associated with them, respectively, with a threshold of FDR < 0.05.

### 2.8. Statistical Analysis

We performed two-class comparisons using Student's *t*-test for normally distributed data. Pearson's or Spearman's correlation test was used to evaluate the correlation between two variables. In analyzing correlations between *TRPV1* expression levels and the enrichment scores of tumor immunosuppressive signatures, tumor stemness, epithelial-mesenchymal transition (EMT), and cancer-related pathways, tumor purity, and stromal scores, we used Spearman's correlation test and reported correlation coefficients (*ρ*). We used Pearson's correlation test, to analyze correlations between *TRPV1* expression levels and the expression levels of a single gene and the ratios of immune signatures, and reported correlation coefficients (*r*). We employed the Benjamini and Hochberg method [28] to calculate the FDR to correct *p* values in multiple tests.

## 3. Results

### 3.1. *TRPV1* Expression Is Negatively Associated with Tumor Proliferation, EMT, Stemness, and Oncogenic Signatures in Cancer

**Figure 1:**
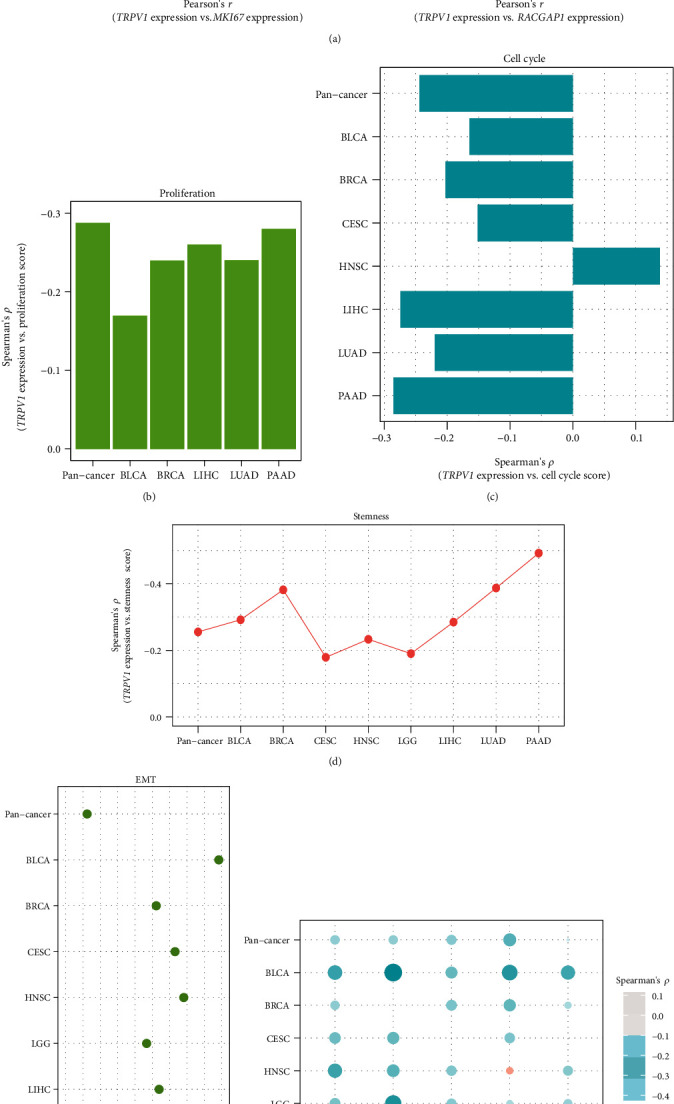
Correlations of *TRPV1* expression with tumor proliferation, stemness, EMT, and oncogenic signaling. Significant negative correlations of *TRPV1* expression levels with *MKI67* expression levels and *RACGAP1* expression levels (a), proliferation scores (b), tumor stemness scores (c), cell cycle scores (d), EMT scores (e), and the enrichment of five oncogenic pathways (f) in pan-cancer and multiple cancer types. Pearson's or Spearman's correlation test *p* value <0.05 indicates a significant correlation; the correlation coefficients are shown. All analyses were performed in 10 cancer types, while only the cancer types in which the results were significant (*p* < 0.05) are shown in the figure.

Sustaining proliferative signaling and enabling replicative immortality are two hallmarks of cancer [29]. Notably, *TRPV1* expression had a negative correlation with the expression of *MKI67*, a marker for cell proliferation [28], in pan-cancer (*p* = 3.04 × 10^−93^; *r* = −0.28) and in five cancer types (*p* < 0.05) (Figure 1(a)). Moreover, *TRPV1* expression correlated negatively with the expression of *RACGAP1*, another marker for cell proliferation [30], in pan-cancer (*p* = 5.80 × 10^−98^; *r* = −0.29) as well as in five cancer types (*p* < 0.001) (Figure 1(a)). We further analyzed the expression correlation of *TRPV1* with a proliferation signature, which involves seven marker genes (*CCNB1*, *CDC20*, *CDKN3*, *CDK1*, *MAD2L1*, *PRC1*, and *RRM2*) [31]. Again, their correlation was significant and negative in pan-cancer (*p* = 2.20 × 10^−98^; *ρ* = −0.29) and in five cancer types (*p* < 0.001) (Figure 1(b)). Likewise, *TRPV1* expression levels also displayed a significant inverse correlation with cell cycle scores in pan-cancer (*p* = 9.75 × 10^−71^; *ρ* = −0.24) and in six cancer types (*p* < 0.01) (Figure 1(c)).

Tumor stemness indicates the stem cell-like feature shown in certain tumor cells that drives cancer advancement, invasion, immunosuppression, and drug resistance [32]. We found significant negative correlations between *TRPV1* expression levels and tumor stemness scores in pan-cancer (*p* = 1.47 × 10^−77^; *ρ* = −0.26) and in eight cancer types (*p* < 0.01) (Figure 1(d)).

EMT has an important role in malignant transformation and tumor progression [33]. Interestingly, *TRPV1* expression levels correlated negatively with the enrichment scores of the EMT signature in nine individual cancer types (*p* < 0.001) (Figure 1(e)). However, in pan-cancer, they showed a positive correlation (*p* = 0.006; *ρ* = 0.04). These results reflect Simpson's paradox [34], an uninformative statistical error.

We also explored the correlations of *TRPV1* expression levels with the enrichment of five cancer-related pathways (p53, mTOR, Wnt, MAPK, and ErbB signaling) in cancer. Our analysis showed that the correlations tended to be negative (*p* < 0.05) (Figure 1(f)).

In summary, our results suggest that *TRPV1* downregulation is associated with unfavorable tumor progression phenotypes in cancer.

### 3.2. *TRPV1* Downregulation Is Associated with Inferior Clinical Outcomes in Cancer

**Figure 2:**
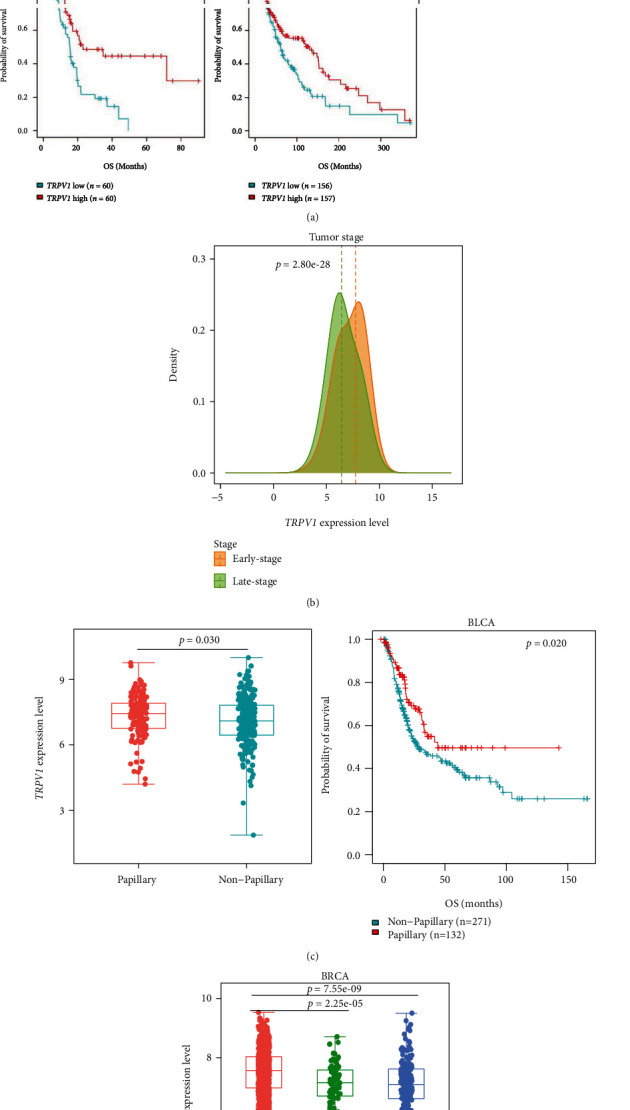
Correlations of *TRPV1* expression with clinical characteristics and cancer subtypes. *TRPV1* expression levels correlate positively with survival prognosis (a) and are lower in late-stage than in early-stage tumors in pan-cancer (b). *TRPV1* expression levels are significantly higher in papillary than in nonpapillary subtypes of BLCA, and the papillary subtype has a better OS than the nonpapillary subtype (c). In BRCA, *TRPV1* expression is significantly lower in basal-like than in luminal A&B and in HER2-enriched than in luminal A&B (d). In LGG, *TRPV1* expression levels are significantly higher in *IDH*-mutated than in *IDH*-wild-type tumors, and the *IDH*-mutated subtype has a better OS than the *IDH*-wild-type subtype (e). OS: overall survival; DSS: disease-specific survival; PFI: progression-free interval. The log-rank test *p* values, the Chi-squared test *p* values, and Student's *t*-test *p* values are shown.

Survival analyses showed a positive correlation between *TRPV1* expression and survival prognosis (OS, DSS, and PFI) in pan-cancer (log-rank test, *p* < 0.001) (Figure 2(a)). Also, in five individual cancer types (BLCA, HNSC, LIHC, PAAD, and SKCM), *TRPV1* downregulation correlated with worse OS (*p* < 0.05) (Figure 2(a)). Moreover, *TRPV1* expression levels were markedly lower in late-stage (stage III-IV) than in early-stage (stage I-II) tumors in pan-cancer (*p* = 2.80 × 10^−28^) (Figure 2(b)).

Furthermore, we compared *TRPV1* expression levels among subtypes of several common cancer types, including BLCA, BRCA, and LGG. In BLCA, *TRPV1* expression levels were markedly higher in papillary than in nonpapillary tumors (*p* < 0.05) (Figure 2(c)). Again, it suggests a positive association between *TRPV1* expression and clinical outcomes in BLCA since the papillary subtype has a better prognosis than the nonpapillary subtype (Figure 2(c)). In BRCA, we compared *TRPV1* expression levels among breast cancer subtypes defined by the PAM50 assay [35]. Notably, *TRPV1* expression levels were significantly lower in basal-like than in luminal A&B (*p* = 7.55 × 10^−9^) and in HER2-enriched than in luminal A&B (*p* = 2.25 × 10^−5^) (Figure 2(d)). These results again indicate that TRPV1 is a positive prognostic factor in breast cancer since basal-like and HER2-enriched subtypes have a worse prognosis than luminal A&B subtypes [36]. In LGG, *TRPV1* expression was remarkably upregulated in *IDH*-mutated versus *IDH*-wild-type tumors (*p* = 0.0002) (Figure 2(e)). Because the *IDH*-mutated subtype has a better OS prognosis compared with the *IDH*-wild-type subtype (Figure 2(e)), it suggests a positive relationship between *TRPV1* expression and clinical outcomes in LGG.

Taken together, our analysis suggests a significant positive association between *TRPV1* expression and clinical outcomes in cancer.

### 3.3. *TRPV1* Expression Is Positively Correlated with Tumor Purity and Negatively Correlated with Stromal Content and Genomic Instability

**Figure 3:**
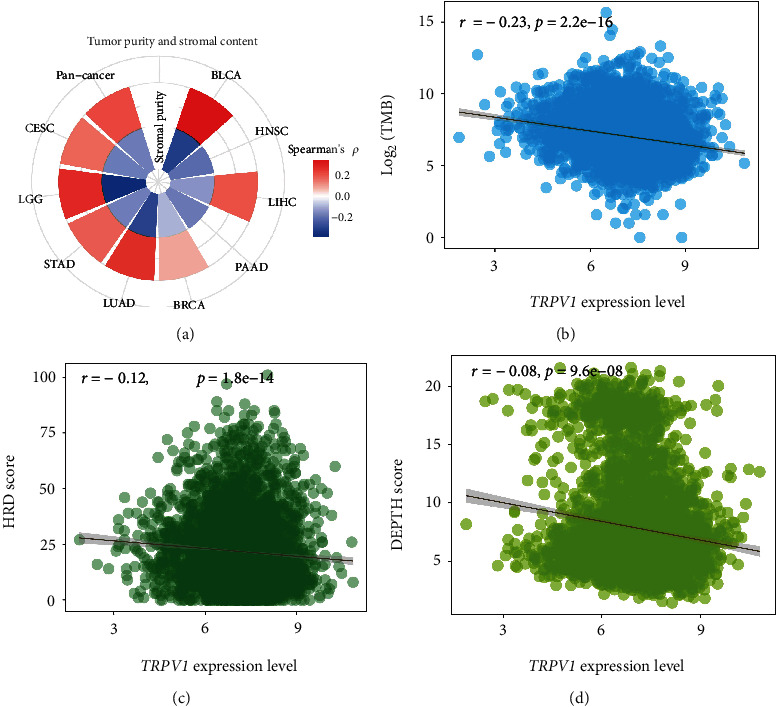
Correlations of *TRPV1* expression with tumor purity, stromal content, TMB, HRD, and DEPTH. The *TRPV1* expression levels correlate positively with tumor purity and correlate negatively with stromal content (a). Significant negative correlations of *TRPV1* expression levels with TMB scores (b), HRD scores (c), and DEPTH scores (d) in pan-cancer. Spearman's correlation test *p* value < 0.05 indicates a significant correlation; the correlation coefficients are shown. All analyses were performed in 10 cancer types, while only the cancer types in which the results were significant (*p* < 0.05) are shown in the figure.

Our analysis revealed that *TRPV1* expression had a marked positive correlation with tumor purity in pan-cancer and seven cancer types (*p* < 0.01), while it showed a significant negative correlation with stromal content in pan-cancer and nine cancer types (*p* < 0.05) (Figure 3(a)).

Genomic instability plays a key role in tumor initiation and progression [37] and often results in increased TMB and tumor aneuploidy [38]. Our analysis showed that *TRPV1* expression levels correlated negatively with TMB in pan-cancer (*p* = 8.92 × 10^−51^; *ρ* = −0.23) (Figure 3(b)). Large-scale genomic instability and tumor aneuploidy are consequences of HRD [26]. Our analysis showed that *TRPV1* expression levels correlated negatively with HRD scores in pan-cancer (*p* = 1.84 × 10^−14^; *ρ* = −0.12) (Figure 3(c)). ITH is a consequence of genomic instability [39] that has a significant association with unfavorable clinical outcomes in cancer [40]. Our analysis revealed a significant negative correlation between *TRPV1* expression levels and ITH scores in pan-cancer (*p* = 9.61 × 10^−8^; *ρ* = −0.081) (Figure 3(d)). These results collectively suggest a negative association between that *TRPV1* expression and genomic instability in cancer.

### 3.4. *TRPV1* Expression Correlated Inversely with Immunosuppressive Signatures in Cancer

**Figure 4:**
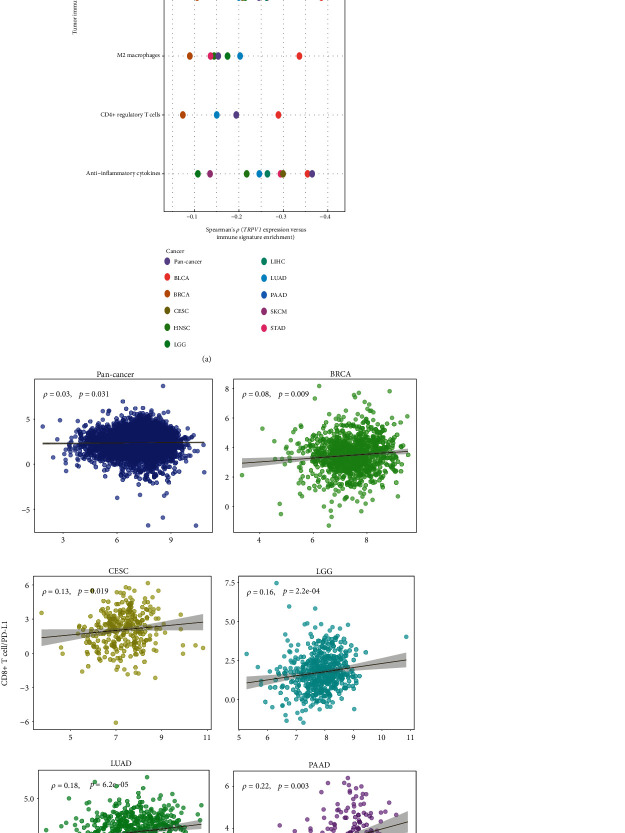
Correlations of *TRPV1* expression with tumor immunosuppressive signature scores and ratios of CD8+ T cell/PD-L1. The significant negative correlation between *TRPV1* expression levels and tumor immunosuppressive signatures' scores (a). *TRPV1* expression levels correlate positively with the ratios of CD8+ T cell/PD-L1 (b). Pearson's or Spearman's correlation test *p* value < 0.05 indicates a significant correlation; the correlation coefficients (*r* or *ρ*) are shown. All analyses were performed in 10 cancer types, while only the cancer types in which the results were significant (*p* < 0.05) are shown in the figure.

Our analysis revealed significant negative correlations between *TRPV1* expression levels and the enrichment scores of numerous immunosuppressive signatures in pan-cancer and in most cancer types (*p* < 0.05) (Figure 4(a)). These immunosuppressive signatures included myeloid-derived suppressor cells (MDSCs), T cell exhaustion, PD-L1, anti-inflammatory cytokines, M2 macrophages, TGF-*β*, and CD4+ regulatory T cells. However, *TRPV1* expression levels had a positive correlation with the ratios of immunostimulatory over immunosuppressive signatures (CD8+ T cell/PD-L1) in pan-cancer and in five cancer types (*p* < 0.05) (Figure 4(b)). These results suggest that TRPV1 may play a role in promoting the antitumor immune response.

### 3.5. Identification of TRPV1-Associated Network and Pathway

**Figure 5:**
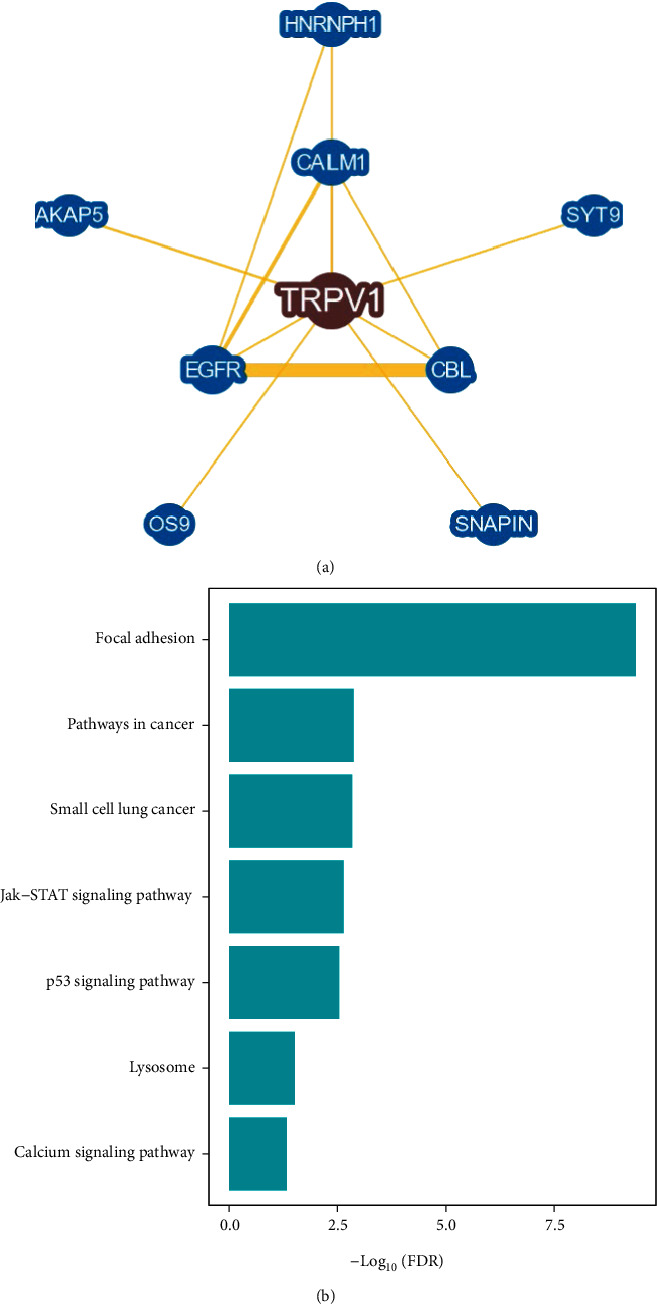
TRPV1 interaction network analysis and pathways significantly associated with *TRPV1* expression. (a) The TRPV1 interaction network and its eight gene interactors identified by BioGRID [41]. (b) Cancer-related pathways whose enrichment shows significant negative correlations with *TRPV1* expression (FDR < 0.05), identified by GSEA [47].

Network analysis by BioGRID [41] uncovered the interaction relationship between TRPV1 and eight proteins/genes (Figure 5(a)). The eight interactors of TRPV1 included CBL, EGFR, CALM1, HNRNPH1, AKAP5, SYT9, OS9, and SNAPIN. Among those interactors, CBL as a proto-oncogene plays an important role in cancer, whose mutations can enhance the PI3K/AKT signaling [42]. EGFR is a tumor driver factor whose overexpression may promote tumor cell proliferation [43]. TRPV1 promotes the ubiquitination of EGFR by the ubiquitin ligase Cbl, leading to the degradation of EGFR through the lysosomal pathway [44]. It indicates that *TRPV1* expression is positively associated with tumor prognosis through multiple mechanisms.

GSEA [26] identified 37 and 29 KEGG pathways significantly associated with the top 500 genes upregulated in the high- and low-*TRPV1*-expression subgroups, respectively. Notably, there were numerous oncogenic pathways upregulated in the low-*TRPV1*-expression subgroup, including pathways in cancer, small cell lung cancer, Jak-STAT signaling, p53 signaling, and calcium signaling (Figure 5(b)). It supports the previous results of the negative association between *TRPV1* expression and the enrichment of oncogenic pathways.

## 4. Discussion

For the first time, we comprehensively analyzed the correlations of *TRPV1* expression levels with tumor proliferation, stemness, EMT, genomic instability, ITH, immunity, and various clinical features in pan-cancer and diverse cancer types. We found that *TRPV1* expression levels correlated negatively with the expression levels of the tumor proliferation index marker *MKI67* and *RACGAP1*, proliferation score, cell cycle score, tumor stemness, EMT, TMB, HRD, ITH, stromal content, tumor immunosuppressive signatures, and oncogenic pathways' enrichment. As a result, *TRPV1* downregulation was associated with unfavorable clinical outcomes in cancer.

Our analysis supports a significant negative correlation between *TRPV1* expression and tumor progression in pan-cancer and multiple individual cancer types. It is in line with previous studies showing that *TRPV1* expression correlates negatively with the expression of cancer proliferation and metastasis-related markers (Ki67 and VEGFR) [18] and that the activation of TRPV1 can significantly inhibit cancer cell growth by inducing apoptosis and necrosis [15]. In fact, a previous study has demonstrated that the use of TRPV1 agonists can promote tumor cell proliferation [45], supporting our findings.

The tumor suppressive effect of TRPV1 may be achieved through multiple pathways. First, TRPV1 can regulate the flow of calcium ions, thereby reducing the proliferation of tumor cells. Previous studies have shown that TRPV1 can inhibit the development of cancer by regulating the Ca^2+^/CaMKK*β*/AMPK pathway [18]. Second, *TRPV1* expression can downregulate the EGFR/MAPK signaling [19], thereby inhibiting the EGFR-induced epithelial cell proliferation [21]. Finally, *TRPV1* expression may promote antitumor immunity. It supports a previous study showing that the tumor suppression role of TRPV1 is associated with its positive correlation with antitumor immune infiltration in ccRCC [20]. Our analysis also demonstrates the positive association between *TRPV1* expression and antitumor immune responses, as evidenced by *TRPV1* expression having a negative association with tumor immunosuppressive signaling and a positive association with the ratio of immunostimulatory to immunosuppressive signatures.

Notably, the p53 pathway is recognized as a tumor suppressor pathway, while our prior study has shown that the tumors highly expressing *TP53* have worse prognosis than the tumors lowly expressing *TP53* [35]. A potential explanation for this could be that tumor progression stimulates the upregulation of the p53 pathway. Therefore, the negative correlation between the expression levels of *TRPV1* and the enrichment scores of the p53 pathway could be attributed to the inhibitory effect of TRPV1 on cancer progression that reduces the stimulatory upregulation of the p53 pathway.

**Figure 6:**
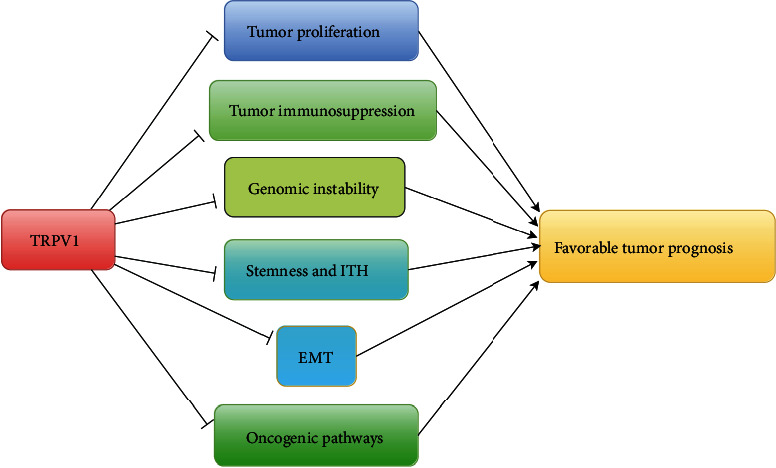
The potential mechanism of *TRPV1* functioning as a tumor suppressor.

Notably, TRPV1 as a pain and heat receptor is often considered a target for pain relief [46]. However, our study suggests that TRPV1 is likely to act as a tumor suppressor (Figure 6). Thus, treating pain with a high dose or long-term usage of TRPV1 inhibitors should be cautious for their potential adverse oncogenic effects.

This research has several limitations. First, our analyses are merely based on bioinformatics analysis but lack of experimental validation. Second, this research used the mRNA expression data to perform all analyses, which may not fully recapitulate the protein expression profiles of TRPV1 in cancer.

## Data Availability

The datasets of RNA-seq, somatic mutations, and clinical information for ten TCGA cancer cohorts can be downloaded from the GDC database (https://portal.gdc.cancer.gov/). The cancer subtype-related data can be downloaded from TCGA with the R function “TCGAquery_subtype” in the R package “TCGAbiolinks”.

## Supplementary Materials

Supplementary Materials Table S1: a description of the datasets for ten TCGA cancer cohorts analyzed in this study. Table S2: the gene sets representing immune signatures, tumor phenotypes, and pathways.
