# Supplementary material for: TRPV1 Is a Potential Tumor Suppressor for Its Negative Association with Tumor Proliferation and Positive Association with Antitumor Immune Responses in Pan-Cancer
Source: J Oncol. 2022 Oct 18;2022:6964550. doi: 10.1155/2022/6964550 (PMC9596243; doi:10.1155/2022/6964550)
Supplement: Supplementary Materials — Table S1: a description of the datasets for ten TCGA cancer cohorts analyzed in this study. Table S2: the gene sets representing immune signatures, tumor phenotypes, and pathways. [file 6964550.f1.docx]

| **Table S1. A summary of the datasets analyzed** | | |
| --- | --- | --- |
| **Cancer** | **Full name** | **Cancer samples** |
| BLCA | Bladder urothelial carcinoma | 408 |
| BRCA | Breast invasive carcinoma | 1100 |
| CESC | Colon adenocarcinoma | 287 |
| HNSC | Head and neck squamous cell carcinoma | 522 |
| LGG | Brain lower grade glioma | 373 |
| LIHC | Liver hepatocellular carcinoma | 517 |
| LUAD | Lung adenocarcinoma | 179 |
| PAAD | Pancreatic adenocarcinoma | 95 |
| SKCM | Skin cutaneous melanoma | 472 |
| STAD | Stomach adenocarcinoma | 509 |
| Pan-cancer |  | 4462 |

**Table S2. Gene sets of immune-related signatures, phenotypes, and cancer-related pathways**

| Tumor aggressive phenotypes | Proliferation | CCNB1, CDC20, CDKN3, CDK1, MAD2L1, PRC1, RRM2 |
| --- | --- | --- |
|  | stemness | DNMT3B, PFAS, XRCC5, HAUS6, TET1, IGF2BP1, PLAA, TEX10, MSH6, DLGAP5, MTREX, SOHLH2, RRAS2, PAICS, CPSF3, LIN28B, IPO5, BMPR1A, ZNF788P, ASCC3, FANCB, HMGA2, TRIM24, ORC1, HDAC2, HESX1, INHBE, MIS18A, DCUN1D5, MRPL3, CENPH, MYCN, HAUS1, GDF3, TBCE, RIOK2, BCKDHB, RAD1, NREP, ADH5, PLRG1, ROR1, RAB3B, DIAPH3, GNL2, FGF2, NMNAT2, KIF20A, CENPI, DDX1, XXYLT1, GPR176, BBS9, RTRAF, BOD1, CDC123, SNRPD3, FAM118B, DPH3, EIF2B3, RPF2, APLP1, DACT1, PDHB, C14orf119, DTD1, SAMM50, CCL26, MED20, UTP6, RARS2, ARMCX2, RARS, MTHFD2, DHX15, HTR7, MTHFD1L, ARMC9, XPOT, IARS, HDX, ACTRT3, ERCC2, TBC1D16, GARS, KIF7, UBE2K, SLC25A3, ICMT, UGGT2, ATP11C, SLC24A1, EIF2AK4, GPX8, ALX1, OSTC, TRPC4, HAS2, FZD2, TRNT1, MMADHC, SNX8, CDH6, HAT1, SEC11A, DIMT1, TM2D2, FST, GBE1 |
|  | EMT | CDH2, SNAI1, TGFB1, ZEB1, ZEB2 |
| Cancer-associated pathways | Cell cycle | ABL1, ANAPC1, ANAPC10, ANAPC11, ANAPC13, ANAPC2, ANAPC4, ANAPC5, ANAPC7, ATM, ATR, BUB1, BUB1B, BUB3, CCNA1, CCNA2, CCNB1, CCNB2, CCNB3, CCND1, CCND2, CCND3, CCNE1, CCNE2, CCNH, CDC14A, CDC14B, CDC16, CDC20, CDC23, CDC25A, CDC25B, CDC25C, CDC26, CDC27, CDC45, CDC6, CDC7, CDK1, CDK2, CDK4, CDK6, CDK7, CDKN1A, CDKN1B, CDKN1C, CDKN2A, CDKN2B, CDKN2C, CDKN2D, CHEK1, CHEK2, CREBBP, CUL1, DBF4, E2F1, E2F2, E2F3, E2F4, E2F5, EP300, ESPL1, FZR1, GADD45A, GADD45B, GADD45G, GSK3B, HDAC1, HDAC2, MAD1L1, MAD2L1, MAD2L2, MCM2, MCM3, MCM4, MCM5, MCM6, MCM7, MDM2, MYC, ORC1, ORC2, ORC3, ORC4, ORC5, ORC6, PCNA, PKMYT1, PLK1, PRKDC, PTTG1, PTTG2, RAD21, RB1, RBL1, RBL2, RBX1, SFN, SKP1, SKP2, SMAD2, SMAD3, SMAD4, SMC1A, SMC1B, SMC3, STAG1, STAG2, TFDP1, TFDP2, TGFB1, TGFB2, TGFB3, TP53, TTK, WEE1, WEE2, YWHAB, YWHAE, YWHAG, YWHAH, YWHAQ, YWHAZ, ZBTB17 |
|  | p53 | ATM, CHEK2, ATR, CHEK1, GORAB, CDKN2A, MDM2, MDM4, TP53, CDKN1A, CCND1, CCND2, CCND3, CDK4, CDK6, CCNE1, CCNE2, CDK2, SFN, RPRM, CCNB1, CCNB2, CDK1, GADD45A, GADD45B, GADD45G, GTSE1, FAS, PIDD1, CASP8, BID, BAX, PMAIP1, BBC3, TP53AIP1, SIVA1, BCL2L1, BCL2, TP53I3, EI24, SHISA5, PERP, ZMAT3, SIAH1, CYCS, APAF1, CASP9, CASP3, AIFM2, IGFBP3, IGF1, SERPINE1, ADGRB1, CD82, THBS1, SERPINB5, DDB2, RRM2B, RRM2, SESN1, SESN3, SESN2, PTEN, TSC2, STEAP3, COP1, RCHY1, CCNG1, CCNG2, PPM1D, TP73, TNFRSF10B |
|  | mTOR | AKT1, AKT1S1, AKT2, AKT3, ATP6V1A, ATP6V1B1, ATP6V1B2, ATP6V1C1, ATP6V1C2, ATP6V1D, ATP6V1E1, ATP6V1E2, ATP6V1F, ATP6V1G1, ATP6V1G2, ATP6V1G3, ATP6V1H, BRAF, CAB39, CAB39L, CASTOR1, CASTOR2, CHUK, CLIP1, DDIT4, DEPDC5, DEPTOR, DVL1, DVL2, DVL3, EIF4B, EIF4E, EIF4E1B, EIF4E2, EIF4EBP1, FLCN, FNIP1, FNIP2, FZD1, FZD10, FZD2, FZD3, FZD4, FZD5, FZD6, FZD7, FZD8, FZD9, GRB10, GRB2, GSK3B, HRAS, IGF1, IGF1R, IKBKB, INS, INSR, IRS1, KRAS, LAMTOR1, LAMTOR2, LAMTOR3, LAMTOR4, LAMTOR5, LPIN1, LRP5, LRP6, MAP2K1, MAP2K2, MAPK1, MAPK3, MAPKAP1, MIOS, MLST8, MTOR, NPRL2, NPRL3, NRAS, PDPK1, PIK3CA, PIK3CB, PIK3CD, PIK3R1, PIK3R2, PIK3R3, PRKAA1, PRKAA2, PRKCA, PRKCB, PRKCG, PRR5, PTEN, RAF1, RHEB, RHOA, RICTOR, RNF152, RPS6, RPS6KA1, RPS6KA2, RPS6KA3, RPS6KA6, RPS6KB1, RPS6KB2, RPTOR, RRAGA, RRAGB, RRAGC, RRAGD, SEC13, SEH1L, SESN2, SGK1, SKP2, SLC38A9, SLC3A2, SLC7A5, SOS1, SOS2, STK11, STRADA, STRADB, TBC1D7, TBC1D7-LOC100130357, TELO2, TNF, TNFRSF1A, TSC1, TSC2, TTI1, ULK1, ULK2, WDR24, WDR59, WNT1, WNT10A, WNT10B, WNT11, WNT16, WNT2, WNT2B, WNT3, WNT3A, WNT4, WNT5A, WNT5B, WNT6, WNT7A, WNT7B, WNT8A, WNT8B, WNT9A, WNT9B |
|  | Wnt | APC, APC2, AXIN1, AXIN2, BAMBI, BTRC, CACYBP, CAMK2A, CAMK2B, CAMK2D, CAMK2G, CBY1, CCN4, CCND1, CCND2, CCND3, CER1, CHD8, CREBBP, CSNK1A1, CSNK1A1L, CSNK1E, CSNK2A1, CSNK2A2, CSNK2A3, CSNK2B, CTBP1, CTBP2, CTNNB1, CTNNBIP1, CTNND2, CUL1, CXXC4, DAAM1, DAAM2, DKK1, DKK2, DKK4, DVL1, DVL2, DVL3, EP300, FBXW11, FOSL1, FRAT1, FRAT2, FZD1, FZD10, FZD2, FZD3, FZD4, FZD5, FZD6, FZD7, FZD8, FZD9, GPC4, GSK3B, INVS, JUN, LEF1, LGR4, LGR5, LGR6, LRP5, LRP6, MAP3K7, MAPK10, MAPK8, MAPK9, MMP7, MYC, NFATC1, NFATC2, NFATC3, NFATC4, NKD1, NKD2, NLK, NOTUM, PLCB1, PLCB2, PLCB3, PLCB4, PORCN, PPARD, PPP3CA, PPP3CB, PPP3CC, PPP3R1, PPP3R2, PRICKLE1, PRICKLE2, PRICKLE3, PRICKLE4, PRKACA, PRKACB, PRKACG, PRKCA, PRKCB, PRKCG, PSEN1, RAC1, RAC2, RAC3, RBX1, RHOA, RNF43, ROCK2, ROR1, ROR2, RSPO1, RSPO2, RSPO3, RSPO4, RUVBL1, RYK, SENP2, SERPINF1, SFRP1, SFRP2, SFRP4, SFRP5, SIAH1, SKP1, SMAD3, SMAD4, SOST, SOX17, TBL1X, TBL1XR1, TBL1Y, TCF7, TCF7L1, TCF7L2, TP53, TPTEP2-CSNK1E, VANGL1, VANGL2, WIF1, WNT1, WNT10A, WNT10B, WNT11, WNT16, WNT2, WNT2B, WNT3, WNT3A, WNT4, WNT5A, WNT5B, WNT6, WNT7A, WNT7B, WNT8A, WNT8B, WNT9A, WNT9B, ZNRF3 |
|  | MAPK | AKT1, AKT2, AKT3, ANGPT1, ANGPT2, ANGPT4, ARAF, AREG, ARRB1, ARRB2, ATF2, ATF4, BDNF, BRAF, CACNA1A, CACNA1B, CACNA1C, CACNA1D, CACNA1E, CACNA1F, CACNA1G, CACNA1H, CACNA1I, CACNA1S, CACNA2D1, CACNA2D2, CACNA2D3, CACNA2D4, CACNB1, CACNB2, CACNB3, CACNB4, CACNG1, CACNG2, CACNG3, CACNG4, CACNG5, CACNG6, CACNG7, CACNG8, CASP3, CD14, CDC25B, CDC42, CHUK, CRK, CRKL, CSF1, CSF1R, DAXX, DDIT3, DUSP1, DUSP10, DUSP16, DUSP2, DUSP3, DUSP4, DUSP5, DUSP6, DUSP7, DUSP8, DUSP9, ECSIT, EFNA1, EFNA2, EFNA3, EFNA4, EFNA5, EGF, EGFR, ELK1, ELK4, EPHA2, ERBB2, ERBB3, ERBB4, EREG, FAS, FASLG, FGF1, FGF10, FGF16, FGF17, FGF18, FGF19, FGF2, FGF20, FGF21, FGF22, FGF23, FGF3, FGF4, FGF5, FGF6, FGF7, FGF8, FGF9, FGFR1, FGFR2, FGFR3, FGFR4, FLNA, FLNB, FLNC, FLT1, FLT3, FLT3LG, FLT4, FOS, GADD45A, GADD45B, GADD45G, GNA12, GNG12, GRB2, HGF, HRAS, HSPA1A, HSPA1B, HSPA1L, HSPA2, HSPA6, HSPA8, HSPB1, IGF1, IGF1R, IGF2, IKBKB, IKBKG, IL1A, IL1B, IL1R1, IL1RAP, INS, INSR, IRAK1, IRAK4, JMJD7-PLA2G4B, JUN, JUND, KDR, KIT, KITLG, KRAS, LAMTOR3, MAP2K1, MAP2K2, MAP2K3, MAP2K4, MAP2K5, MAP2K6, MAP2K7, MAP3K1, MAP3K11, MAP3K12, MAP3K13, MAP3K14, MAP3K2, MAP3K20, MAP3K3, MAP3K4, MAP3K5, MAP3K6, MAP3K7, MAP3K8, MAP4K1, MAP4K2, MAP4K3, MAP4K4, MAPK1, MAPK10, MAPK11, MAPK12, MAPK13, MAPK14, MAPK3, MAPK7, MAPK8, MAPK8IP1, MAPK8IP2, MAPK8IP3, MAPK9, MAPKAPK2, MAPKAPK3, MAPKAPK5, MAPT, MAX, MECOM, MEF2C, MET, MKNK1, MKNK2, MRAS, MYC, MYD88, NF1, NFATC1, NFATC3, NFKB1, NFKB2, NGF, NGFR, NLK, NR4A1, NRAS, NTF3, NTF4, NTRK1, NTRK2, PAK1, PAK2, PDGFA, PDGFB, PDGFC, PDGFD, PDGFRA, PDGFRB, PGF, PLA2G4A, PLA2G4B, PLA2G4C, PLA2G4D, PLA2G4E, PLA2G4F, PPM1A, PPM1B, PPP3CA, PPP3CB, PPP3CC, PPP3R1, PPP3R2, PPP5C, PPP5D1, PRKACA, PRKACB, PRKACG, PRKCA, PRKCB, PRKCG, PTPN5, PTPN7, PTPRR, RAC1, RAC2, RAC3, RAF1, RAP1A, RAP1B, RAPGEF2, RASA1, RASA2, RASGRF1, RASGRF2, RASGRP1, RASGRP2, RASGRP3, RASGRP4, RELA, RELB, RPS6KA1, RPS6KA2, RPS6KA3, RPS6KA4, RPS6KA5, RPS6KA6, RRAS, RRAS2, SOS1, SOS2, SRF, STK3, STK4, STMN1, TAB1, TAB2, TAOK1, TAOK2, TAOK3, TEK, TGFA, TGFB1, TGFB2, TGFB3, TGFBR1, TGFBR2, TNF, TNFRSF1A, TP53, TRADD, TRAF2, TRAF6, VEGFA, VEGFB, VEGFC, VEGFD |
|  | ErbB | ABL1, ABL2, AKT1, AKT2, AKT3, ARAF, AREG, BAD, BRAF, BTC, BUB1B-PAK6, CAMK2A, CAMK2B, CAMK2D, CAMK2G, CBL, CBLB, CDKN1A, CDKN1B, CRK, CRKL, EGF, EGFR, EIF4EBP1, ELK1, ERBB2, ERBB3, ERBB4, EREG, GAB1, GRB2, GSK3B, HBEGF, HRAS, JUN, KRAS, MAP2K1, MAP2K2, MAP2K4, MAP2K7, MAPK1, MAPK10, MAPK3, MAPK8, MAPK9, MTOR, MYC, NCK1, NCK2, NRAS, NRG1, NRG2, NRG3, NRG4, PAK1, PAK2, PAK3, PAK4, PAK5, PAK6, PIK3CA, PIK3CB, PIK3CD, PIK3R1, PIK3R2, PIK3R3, PLCG1, PLCG2, PRKCA, PRKCB, PRKCG, PTK2, RAF1, RPS6KB1, RPS6KB2, SHC1, SHC2, SHC3, SHC4, SOS1, SOS2, SRC, STAT5A, STAT5B, TGFA |
| Immune signatures | CD8+ T cells | CD8A |
|  | PD-L1 | CD274 |
|  | CD4+ regulatory T cells | CTLA4, FOXP3, GPR15, IL32, IL4, IL5 |
|  | M2 macrophages | CCL22, FCER2, MRC1, TGM2 |
|  | Anti-inflammatory cytokines | IL10, IL11, IL4, TGFB1 |
|  | TGF-β | ACVR1, ACVR1B, ACVR1C, ACVR2A, ACVR2B, AMH, AMHR2, BAMBI, BMP2, BMP4, BMP5, BMP6, BMP7, BMP8A, BMP8B, BMPR1A, BMPR1B, BMPR2, CDKN2B, CHRD, CREBBP, CUL1, DCN, E2F4, E2F5, EP300, FBN1, FMOD, FST, GDF5, GDF6, GDF7, GREM1, GREM2, HAMP, HJV, ID1, ID2, ID3, ID4, IFNG, INHBA, INHBB, INHBC, INHBE, LEFTY1, LEFTY2, LTBP1, MAPK1, MAPK3, MICOS10-NBL1, MYC, NBL1, NEO1, NODAL, NOG, PITX2, PPP2CA, PPP2CB, PPP2R1A, PPP2R1B, RBL1, RBX1, RGMA, RGMB, RHOA, ROCK1, RPS6KB1, RPS6KB2, SKP1, SMAD1, SMAD2, SMAD3, SMAD4, SMAD5, SMAD6, SMAD7, SMAD9, SMURF1, SMURF2, SP1, TFDP1, TGFB1, TGFB2, TGFB3, TGFBR1, TGFBR2, TGIF1, TGIF2, THBS1, THSD4, TNF, ZFYVE16, ZFYVE9 |
|  | T cell exhaustion | CTLA4, CXCL13, HAVCR2, LAG3, LAYN, PDCD1, TIGIT |
|  | MDSCs | CCR2, CD14, CD2, CD86, CXCR4, FCGR2A, FCGR2B, FCGR3A, FERMT3, GPSM3, IL18BP, IL4R, ITGAL, ITGAM, PARVG, PSAP, PTGER2, PTGES2, S100A8, S100A9 |
